# Hot Water Extract of *Sasa borealis* (Hack.) Makino & Shibata Abate Hydrogen Peroxide-Induced Oxidative Stress and Apoptosis in Kidney Epithelial Cells

**DOI:** 10.3390/antiox11051013

**Published:** 2022-05-20

**Authors:** Ilekuttige Priyan Shanura Fernando, Kirinde Gedara Isuru Sandanuwan Kirindage, Arachchige Maheshika Kumari Jayasinghe, Eui Jeong Han, Mawalle Kankanamge Hasitha Madhawa Dias, Kyung Pil Kang, Sung Ig Moon, Tai Sun Shin, Ayeong Ma, Kyungsook Jung, Ginnae Ahn

**Affiliations:** 1 Department of Marine Bio-Food Sciences, Chonnam National University, Yeosu 59626, Korea; shanura@chonnam.ac.kr; 2 Department of Food Technology and Nutrition, Chonnam National University, Yeosu 59626, Korea; 218388@jnu.ac.kr (K.G.I.S.K.); 218385@jnu.ac.kr (A.M.K.J.); iosu5772@jnu.ac.kr (E.J.H.); 3 College of Veterinary Medicine, Chungnam National University, Yuseong-gu, Daejeon 34134, Korea; hasithadias@o.cnu.ac.kr; 4 Jeju Changhae Fisheries Co., Ltd., Jeju 63072, Korea; golf87@naver.com (K.P.K.); jejuchanghae@naver.com (S.I.M.); 5 Department of Food Science and Nutrition, Chonnam National University, 77 Yongbong-ro, Buk-gu, Gwangju 61186, Korea; shints@chonnam.ac.kr (T.S.S.); ayeong_ma@daum.net (A.M.); 6 Functional Biomaterials Research Center, Korea Research Institute of Bioscience and Biotechnology, 181 Ipsin-gil, Jeongup-si 56212, Korea

**Keywords:** *Sasa borealis*, Vero cells, antioxidants, polyphenols, liver injury, hydrogen peroxide

## Abstract

*Sasa borealis* (Hack.) Makino & Shibata or broad-leaf bamboo is famous for its richness of bioactive natural products and its uses in traditional medicine for its anti-inflammatory, diuretic, and antipyretic properties and preventive effects against hypertension, arteriosclerosis, cardiovascular disease, and cancer. The present study investigated the antioxidant activity of *S. borealis* hot water extract (SBH) and its effects in ameliorating hydrogen peroxide-induced oxidative stress, using an African green monkey kidney epithelial cell line (Vero). Known polyphenols in SBH were quantified by HPLC analysis. SBH indicated a dose-dependent increase for reducing power, ABTS^+^ (IC_50_ = 96.44 ± 0.61 µg/mL) and DPPH (IC_50_ = 125.78 ± 4.41 µg/mL) radical scavenging activities. SBH markedly reduced intracellular reactive oxygen species (ROS) generation in the Vero cells and increased the protective effects against H_2_O_2_-induced oxidative stress by reducing apoptosis. Other than the direct involvement in neutralizing ROS, metabolites in SBH were also found to induce NRF2-mediated production of antioxidant enzymes, HO-1, and NQO1. These findings imply that *S. borealis* hot water extract can be utilized to create nutraceutical and functional foods that can help to relieve the effects of oxidative stress in both acute and chronic kidney injury.

## 1. Introduction

The kidney is a vulnerable target organ for oxidative cellular damage. Oxidative stress has been identified as a major exacerbating factor in both acute and chronic kidney injuries. Studies indicate that the vasoreactivity and myogenic response in kidneys are influenced by reactive oxygen species (ROS) such as superoxide (O_2_^−^) and nitric oxide (NO) [1]. Vero cells derived from the kidney epithetical of an African green monkey are frequently used in biological studies for toxins, viruses, and therapeutic agents [2,3].

*Sasa* (Poaceae) is a genus of perennial grasses, widely distributed in Asian countries such as Korea, Japan, and China. Several species of this genera including *Sasa borealis* (Hack.) Makino & Shibata, also known as broad-leaf bamboo or 조릿대 (jolisdae in Korean) have been used in traditional medicine for their anti-inflammatory, diuretic, and antipyretic properties, and therapeutic effects against hypertension, arteriosclerosis, cardiovascular disease, and cancer [4,5,6,7,8]. The immature leaves of *S. borealis* are marketed as bamboo tea in Korea for their richness of bioactive natural products. The above bioactivities are attributable to numerous polyphenols found in *S. borealis* [4]. Seasonal fluctuations in light intensity alter *S. borealis* leaf area indices and photosynthesis. The biomass of *S. borealis* has been found to decrease with the increasing light intensity while retaining the leaf area index [9]. The young leaves of *S. borealis* are marketed as bamboo tea and it is enriched with antioxidant phytochemicals [10]. Findings by Jeong et al. (2008) indicate that the *S. borealis* leaves are a rich source of proteins, crude fiber, and essential minerals, while its hot water extract contains 12.03% of polyphenols, which contributes to the antioxidant activities confirmed by DPPH and ABTS^+^ radical scavenging assays [11]. In another study, hot water and 95% ethanol extracts of *S. borealis* roots showed prominent DPPH activity, and tyrosinase inhibitory activity in NIH3T3 cells [12].

Information is lacking on the effects of *S. borealis* dietary supplementation on renal cell oxidative stress. Based on the findings that *S. borealis* is a rich source of antioxidant phytochemicals, the present study investigates the effects of *S. borealis* hot-water extract hypothesizing that it would exert cytoprotective effects on hydrogen peroxide-induced oxidative stress in Vero cells.

## 2. Materials and Methods

### 2.1. Materials

*S. borealis* was purchased from Gapdang Herb Co., Ltd. Dongdaemun-gu, Seoul, South Korea. Dimethyl sulfoxide (DMSO), 3-(4,5-Dimethyl-2-thiazolyl)-2,5-diphenyl-2H-tetrazolium bromide (MTT), 2,2-Diphenyl-1-picrylhydrazyl (DPPH), Fluorescein Sodium, 6-Hydroxy-2,5,7,8-tetramethylchroman-2-carboxylic acid (Trolox), 2,2-azino-bis(3-ethylbenzene-thiazoline-6-sulfonic acid) diammonium salt (ABTS^+^), 2,2′-azobis(2-amidino-propane) dihydrochloride (AAPH), 2′,7′-Dichlorofluorescin diacetate (DCFH-DA), Folin–Ciocalteu’s phenol reagent, bovine serum albumin (BSA), ethidium bromide, agarose, propidium iodide (PI), and gallic acid, were bought from Sigma-Aldrich (ST. Louis, MO, USA). Dulbecco’s modified eagle medium (DMEM) and a mixture of antibiotics streptomycin and penicillin (P/S) were purchased from GibcoBRL (Grand Island, NY, USA). Fetal bovine serum (FBS) was obtained from Welgene (Gyeongsangbuk-do, South Korea). Alexa Fluor^®^ 488 conjugated Anti-Mouse IgG was purchased from Thermo Fisher Scientific (Waltham, MA, USA). NE-PER^®^ nuclear and cytoplasmic extraction kit, Pierce™ RIPA buffer, BCA protein assay kit, transfer buffer, polyvinylidene fluoride (PVDF) membranes, and SuperSignal™ West Femto Maximum Sensitivity Substrate, and PageRuler™ Plus pre-stained protein ladder were obtained from Thermo Scientific (Rockford, IL, USA). Antibodies for Western blot analysis, normal goat serum, ProLong, and Phalloidin^®^ Gold antifade containing DAPI, were from Cell Signaling Technology Inc. (Beverly, MA, USA) and Santa Cruz Biotechnology Inc. (Dallas, TX, USA). All the other chemicals and reagents used were of analytical grade. 

### 2.2. Extraction

Dried *S. borealis* leaves were segmented into smaller pieces and ground into a fine powder using an MF10 laboratory grinder (IKA^®^, Staufen, Germany). Each extraction was performed for 4 h in boiling deionized water containing 10% of the sample powder. Extract (SBH) was recovered by centrifugation and vacuum filtration. The filtrate was frozen, lyophilized, and stored at −20 °C in an airtight container. 

### 2.3. Analysis of the Proximate Composition of Major Constituents

Total protein content was measured by Lowry’s method using BSA as the reference standard (Mæhre et al., 2018). The total polyphenol content was determined according to the method of Singleton et al. (1999), using gallic acid as the reference standard [13]. Polysaccharide content was determined by the phenol-sulfuric assay, using d-glucose as the reference standard [14].

### 2.4. Analysis of Antioxidant Activities

DPPH, ABTS^+^ radical scavenging activities, oxygen radical absorbance capacity (ORAC), and reducing power assay (FRAP) were conducted, according to the methods described in our previous publication. Absorbance measurements were taken using a SpectraMax M2 microplate reader (Molecular Devices, CA, USA). 

### 2.5. Cell Culture

Vero cells were obtained from the Korean Cell Line Bank (Seoul, Korea). Cells were cultured in RPMI media supplemented with 10% FBS and 1% antibiotic mix and maintained at 37 °C in a humidified atmosphere with 5% of CO_2_. Sub-culturing was carried out once every 3 days and cells under exponential growth were seeded for experiments. Cells were counted using a hemocytometer after staining with Trypan Blue using a Leica DMIL-LED inverted microscope (Leica, Solms, Germany). The cell suspension was diluted to the desired concentration based on the cell count and used for seeding.

### 2.6. Analysis of Cell Viability and Intracellular ROS Level

Cells were seeded at 1 × 10^4^ cells/mL concentration in 96-well plates for 24 h. Wells were treated with a range of concentrations and incubated for 24 h. Cell viability was evaluated by MTT assay [15]. Absorbance was measured at 570 nm using a SpectraMax M2 microplate reader. To evaluate the protective effects against H_2_O_2_-induced oxidative stress, 24 h pre-seeded culture plates were treated with different concentrations of SBH. Then, 10 μL of H_2_O_2_ (1 mM) was added and incubated at 37 °C for 24 h. Cells were then subjected to MTT assay. The absorbance of formazan crystals dissolved in DMSO was measured by using a SpectraMax M2 microplate reader at 570 nm. The effect of SBH on intracellular ROS levels in H_2_O_2-_induced Vero cells was measured by 2′,7′-dichlorofluorescein diacetate (DCF-DA) assay. In brief, seeded cells were incubated for 16 h and then treated with a series of concentrations of SBH for one hour. Then, H_2_O_2_ (1 mM final concentration) was added to each well and incubated for another 24 h. Finally, intracellular ROS levels were determined by adding 25 μg/mL DCF-DA followed by the emission reading using a microplate reader, and images were captured by using Invitrogen™ EVOS™ Auto 2 fluorescence microscope (Thermo Fisher Scientific, Bothell, WA, USA) [15].

### 2.7. Evaluation of Apoptotic Body Formation

Seeded cells, (1 × 10^5^ cells/well) in 24 well plates after 24 h were treated with different concentrations of SBH for 1 h. The wells apart from the control were stimulated with H_2_O_2_ (1 mM) and incubated for another 24 h. Hoechst 33342 (10 μg/mL) was then added and incubated for 10 min. Afterward, the culture media was removed and new media containing PI was added to the wells and visualized using the EVOS FL Auto 2 Imaging microscope (ThermoFisher Scientific, Waltham, CA, USA) [15]. 

### 2.8. Cell Cycle Analysis

After seeding and treatment, the cells were incubated for 24 h and harvested by trypsinization. Cells were washed with phosphate-buffered saline (PBS), and the cell pellet was obtained by centrifugation at 100× *g* for 5 min. Cells were resuspended in 70% ethanol and allowed to stand at room temperature for 30 min to allow membrane permeabilization. The cells were then washed with a solution of PBS containing EDTA, RNase, and PI. Cell cycle analysis was conducted using a CytoFLEX flow cytometer (Beckman Coulter, PA, USA) [15]. 

### 2.9. Mitochondrial Depolarization Analysis by JC-1 Assay

Mitochondria depolarization is an early event of apoptosis associated with the mitochondria-mediated pathway [16]. After seeding, sample treatment, and stimulation, the cells were incubated for 24 h and harvested by trypsinization. The MitoProbe™ JC-1assay kit (Invitrogen, Carlsbad, CA, USA) was used to evaluate mitochondrial health, following the manufacturers’ instructions. Outcomes were evaluated both by FACS analysis and fluorescence microscopy.

### 2.10. Western Blot Analysis

Intracellular signaling molecules that regulate mitochondria-mediated apoptosis were evaluated by Western blot analysis. The seeded cells, (1 × 10^6^ cells/well) in 6 well plates, after 24 h were treated with different concentrations for 1 h, and the wells except the control were stimulated with H_2_O_2_ (1 mM) followed by 24 h incubation period. Cells were harvested by trypsinization, washed, and lysed using RIPA buffer. The protein content in the lysates was measured by the BCA protein assay kit. Lysates containing 50 μg of proteins were resolved on a polyacrylamide gel alongside the protein ladder by electrophoresis and transferred onto PVDF membranes. Membranes were suspended in Ponceau S solution, washed with deionized water, and cut into strips based on the molecular weight of the intended antibodies. Membrane strips were blocked in Tris-buffered saline with Tween 20 containing 5% skim milk and, respectively, incubated in primary and secondary antibodies. Proteins were visualized by adding SuperSignal™ West Femto Maximum Sensitivity Substrate on a Da Vinci Chemi imaging system (Core Bio, Seoul, Korea).

### 2.11. Immunostaining

After 24 h, the cells seeded in chamber slides (1 × 10^4^ cells/chamber) were treated with different concentrations of SBH for 1 h. Chambers except for the control were stimulated with H_2_O_2_ (1 mM) for a 30 min incubation period. The analysis protocol is described in our previous publication [17]. Images were acquired by an EVOS FL Auto 2 Imaging microscope.

### 2.12. High-Performance Liquid Chromatography (HPLC) Analysis

Analysis was carried out on a Shimadzu HPLC consisting of a CBM-20A system controller, CTO-20AC column oven, SPD-M20A photodiode array detector, and a RID-10A refractive index detector. The separation was carried out on a Luna PFP(2) 100A (150 × 3.0 mm, 3 μm) column. Compounds in SBH were separated using a gradient elution program with a mobile phase consisting of 0.1% formic acid in methanol (A) and 0.1% formic acid in water (B) at a flow rate of 0.34 mL/min. The elution program was applied as follows: 80% A (20% B) decreased to 35% A from 0 to 110 min; decreased to 0% A from 110 to 112 min, and kept at 0% A for 38 min; increased to 100% A from 130 to 132 min, and kept at 100% A for 16 min. Eluants were detected at 270 nm wavelength. A mixture of phenolic compounds with known concentrations was used as the reference standard for the quantification by peak-area measurement. Analysis was carried out in triplicate and the results represent mean ± standard deviation.

### 2.13. Statistical Analysis

Experiments were carried out in triplicate and results are expressed as mean ± standard deviation. The raw data followed a normal distribution, and a variance test revealed that all groups had equal variance. The mean values of each group were compared using the One-way ANOVA test by PASW Statistics 19.0 (Chicago, IL, USA). Mean values with a significant difference at *p* < 0.05 are represented with different letters, as analyzed by Duncan’s multiple range test.

## 3. Results

### 3.1. Yield and Proximate Composition of SBH

The hot water extraction yield of *S. borealis* was 10.83 ± 0.44% (Table 1). Proximate compositional analysis of SBH indicated a higher quantity of polysaccharides (56.41 ± 0.02%), followed by 33.09 ± 0.53% of protein, and 4.82 ± 0.25% of polyphenols.

### 3.2. Composition of Polyphenols in SBH

**Figure 1:**
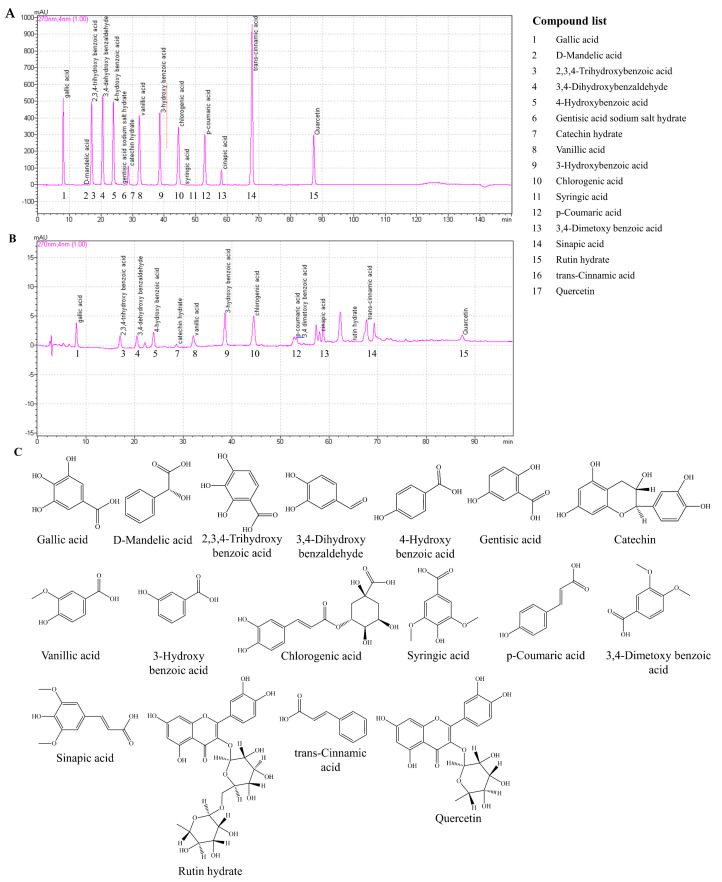
HPLC composition analysis of polyphenols in SBH. Chromatogram of (**A**) flavonoid reference standard and (**B**) SBH. Compounds in SBH were separated using a gradient elution program with a mobile phase consisting of 0.1% formic acid in methanol (solvent A) and 0.1% formic acid in water (solvent B) at a flow rate of 0.34 mL/min; (**C**) Chemical structures of the polyphenols found in SBH.

Phenolic compounds in SBH were quantified by HPLC analysis (Table 2). Seventeen phenolic compounds, those generally found in plants, were used as reference standards (Figure 1). Out of the 17 tested, SBH indicated the presence of 14 compounds, among which quercetin was found in a higher yield.

Figure 1A,B represent the HPLC analysis of polyphenols in SBH in comparison to a standard mixture of known phenolic compounds. Except for the peaks observed at retention times 22.25, 53.28, 50.73, 50.82, and 69.25 min, all of the other peaks were comparable with the retention times in the standard mixture. The structures of the polyphenol compounds are given in Figure 1C.

### 3.3. SBH Indicated Moderate Antioxidant Activity

**Figure 2:**
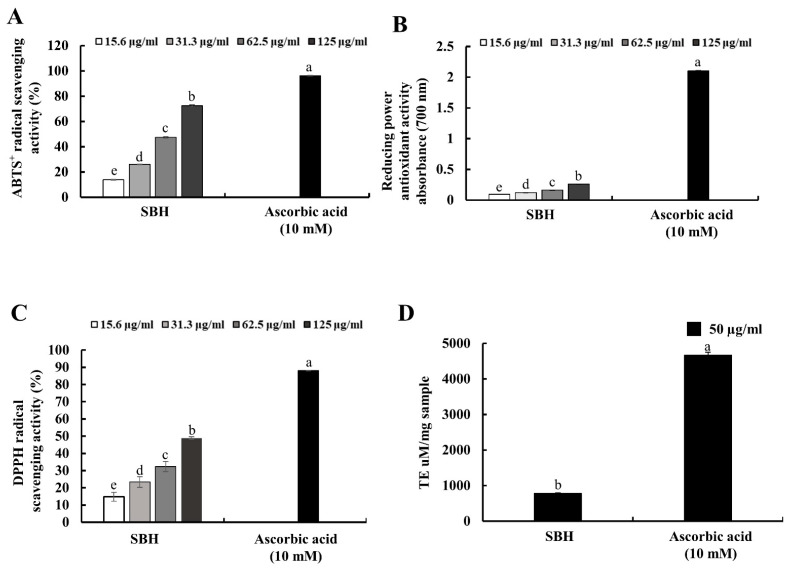
Antioxidant activities of SBH. (**A**) ABTS^+^ radical scavenging; (**B**) reducing power; (**C**) DPPH radical scavenging; and (**D**) oxygen radical absorbance capacity of SBH. Experiments were carried out in triplicate and results were represented as means ± standard deviation (SD) (*n* = 3). Means denoted by different letters are significantly different at *p* < 0.05.

Antioxidant activity of SBH was examined by ABTS^+^ and DPPH radical scavenging activity assays, oxygen radical absorbance capacity (ORAC), and ferric reducing antioxidant power (FRAP) assays compared to the positive control, ascorbic acid (Figure 2). SBH indicated a dose-dependent increase for FRAP, ABTS^+^, and DPPH radical scavenging activities with IC_50_ values of 96.44 ± 0.61, and 125.78 ± 4.41 µg/mL for ABTS^+^ and DPPH radical scavenging activities. When compared with the positive control, ascorbic acid, the antioxidant activity of SBH per ABTS^+^ and DPPH radical scavenging activity could be considered moderate. The ORAC of SBH at 125 μg/mL was 786.21 µmol Trolox equivalents/mg.

### 3.4. SBH Abate Oxidative Stress in H_2_O_2_ Stimulated Vero Cells

**Figure 3:**
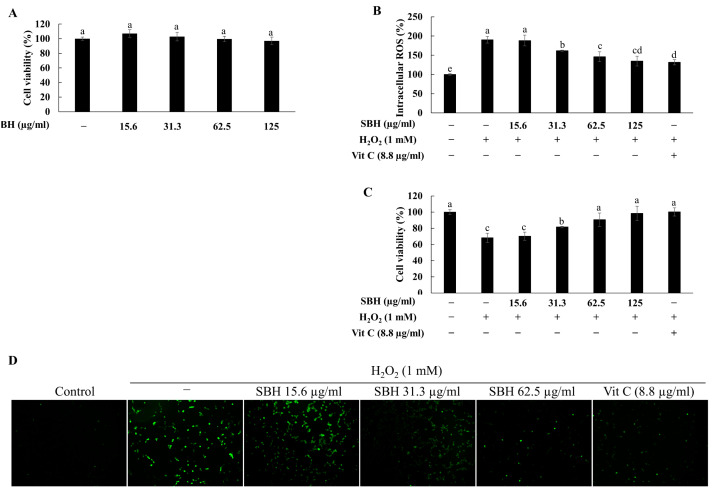
Cytocompatibility and protective effects of SBH against H_2_O_2_-induced oxidative stress in Vero cells. (**A**) Effects of SBH on Vero cell viability; Effects of SBH against H_2_O_2_-induced (**B**) intracellular ROS levels and (**C**) cell viability; (**D**) SBH’s effects on H_2_O_2_-induced intracellular ROS levels as studied by fluorescence microscopy. Experiments were carried out in triplicate and results were represented as means ± SD (*n* = 3). Means denoted by different letters are significantly different at *p* < 0.05.

SBH doses within 15.6–125 µg/mL were not cytotoxic to the Vero cells (Figure 3A). Hence, these concentrations were selected for further experiments. As shown in Figure 3B,C, H_2_O_2_ stimulation increased the intracellular ROS level and reduced the Vero cells viability, compared to the control. SBH-treatment dose-dependently reduced the intracellular ROS level in H_2_O_2_ stimulated cells and increased cell viability, indicating its protective effects. A significant reduction in intracellular ROS level and an increase in cell viability were observed at the SBH dose of 125 μg/mL. The bioactivity of SBH at 125 μg/mL was comparable with the positive control, ascorbic acid, at 50 µM. Further confirmation of SBH’s antioxidant potential was carried out by fluorescence microscopy analysis (Figure 3D), which indicated that dose-dependent SBH treatment would suppress intracellular ROS generation in Vero cells induced by H_2_O_2_ treatment.

### 3.5. SBH Lessens Mitochondrial Depolarization and Apoptosis in H_2_O_2_ Stimulated Vero Cells

**Figure 4:**
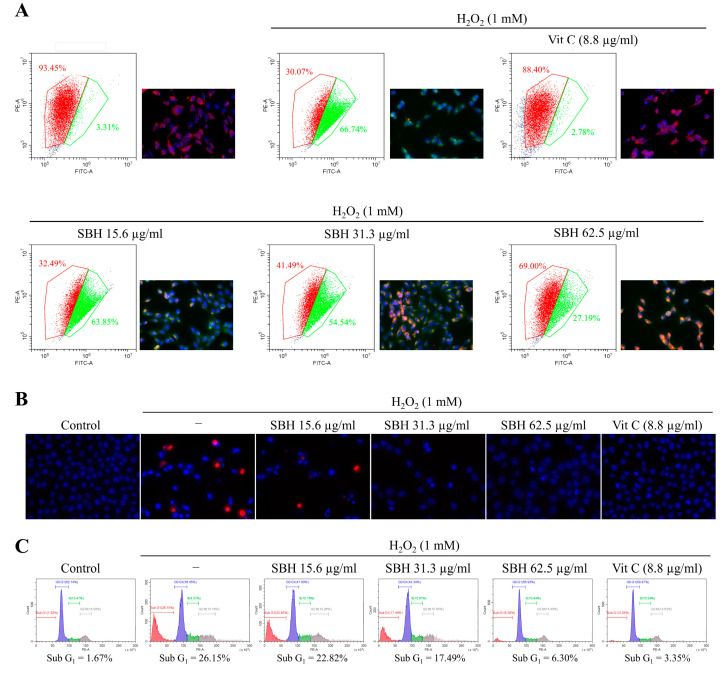
Effects of SBH in reducing mitochondrial depolarization and anti-apoptotic effects in H_2_O_2_-induced Vero cells. (**A**) Effects of SBH against H_2_O_2_-induced mitochondrial depolarization; (**B**) Effects of SBH against H_2_O_2_-induced apoptotic body formation; and (**C**) reduction of the apoptotic hyperdiploid cell population. Experiments were carried out in triplicate (*n* = 3) to ensure repeatability.

Based on the antioxidant and cytoprotective effects observed for SBH, further testing was carried out to evaluate its anti-apoptotic effects in H_2_O_2_-stimulated Vero cells. JC-1 dye is commonly employed to assess mitochondrial health because of its potential-dependent accumulation in mitochondria. At low membrane potential, JC-1 produces green fluorescence whereas at high potential, the dye aggregates and produces red fluorescence [16]. As illustrated in Figure 4A, unhealthy mitochondria (lower red and higher green) were identified in the H_2_O_2_-treated cells. In the SBH-treated cells’ mitochondria, the membrane potential increased dose-dependently, as evidenced by a rise in the red and a reduction in the green emissions. According to Figure 4B, the H_2_O_2_ treatment increased the formation of apoptotic bodies as seen from nuclear condensation and fragmentation per Hoechst 33342 staining, as well as red color fluorescence per PI staining which are indicative of cells undergoing necrosis. Dose-dependent SBH treatment reduced apoptotic body formation. SBH dose of 62.5 μg/mL indicated comparable antiapoptotic activity with the positive control ascorbic acid at 50 µM. Cell cycle analysis was carried out to evaluate the proportion of Sub-G_1_ hypodiploid cells. According to Figure 4C, the H_2_O_2_-treated group had 21.15% of cells under the Sub-G_1_ phase compared to the control (1.62%). Dose-dependent treatment of SBH reduced the proportion of the Sub-G_1_ cells. At an SBH dose of 62.5 μg/mL, the proportion of Sub-G_1_ cells was 6.30%. The positive control ascorbic acid at 50 µM indicated a Sub-G_1_ cell population of 3.35%.

### 3.6. Protective Effects of SBH in H_2_O_2_ Stimulated Vero Cells Are Mediated through Mitochondria-Mediated Apoptosis Pathway

**Figure 5:**
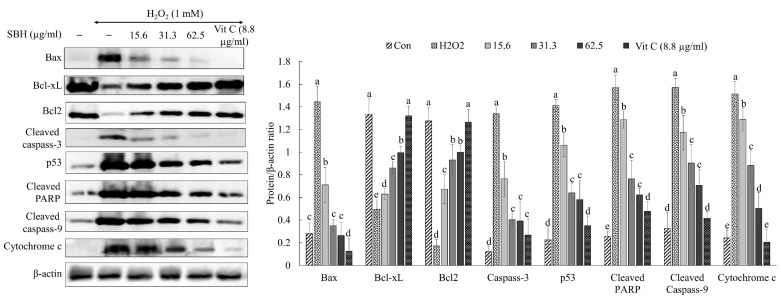
Effects of SBH in mitochondria-mediated apoptosis in H_2_O_2_-induced Vero cells. Experiments were carried out in triplicate and results were represented as means ± SD (*n* = 3). Means denoted by different letters are significantly different at *p* < 0.05.

Apoptosis driven by the mediation of mitochondria is a primary pathway of oxidative stress-induced cell death. In H_2_O_2_-treated cells, cytochrome c, Bax, caspase-3, cleaved caspase-9, cleaved PARP, and p53 levels indicated an increased production, whereas a decrease was seen for anti-apoptotic proteins Bcl2 and Bcl-xL levels (Figure 5). The H_2_O_2_treatment caused PARP cleavage, as evidenced by lower PARP levels and higher cleaved PARP bands. Following increasing dosages of SBH therapy, the levels of apoptotic molecular mediators were dose-dependently reduced, demonstrating SBH’s protective properties against H_2_O_2_-induced oxidative stress.

### 3.7. SBH Increased the Activation of the Nrf2/HO-1/NQO1 Signaling Pathway in H_2_O_2_ Induced Vero Cells

**Figure 6:**
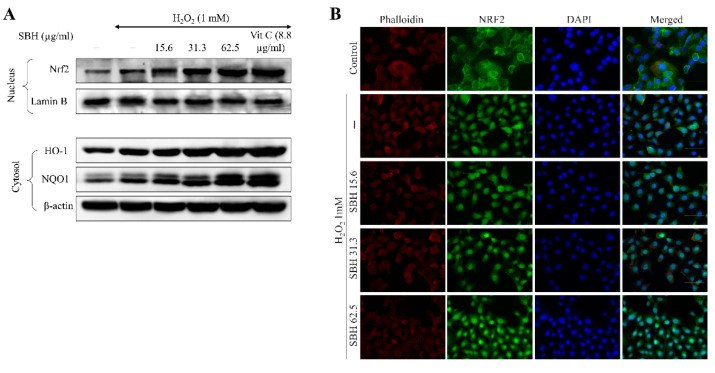
Effects of SBH in increasing the activation of Nrf2/HO-1/NQO1 signaling pathway in H_2_O_2_-induced Vero cells. (**A**) Western blot analysis and (**B**) immunofluorescence analysis of Nrf2 nuclear translocation. Experiments were carried out in triplicate (*n* = 3) to ensure repeatability.3.8. SBH suppressed NF-κB and MAPK signaling in H_2_O_2_-induced Vero cells.

Nrf2 is a key transcription factor that regulates the expression of several antioxidant genes, including HO-1, and NQO1. As shown in Figure 6A, SBH treatment enhanced the baseline levels of Nrf2 in a dose-dependent manner when compared to the H_2_O_2_-treated cells, demonstrating its stimulatory actions. The dose-dependent administration of SBH resulted in a comparable rise in HO-1 and NQO1 levels. Per the immunofluorescence analysis (Figure 6B), the nuclei of cells did not indicate a prominent green fluorescence in the control group, indicating that the Nrf2 is located mainly in the cytosol. The H_2_O_2_ treatment caused Nrf2 nuclear translocation indicated by the increase of green fluorescence within the nucleus, whereas it was further increased with dose-dependent treatment of SBH.

**Figure 7:**
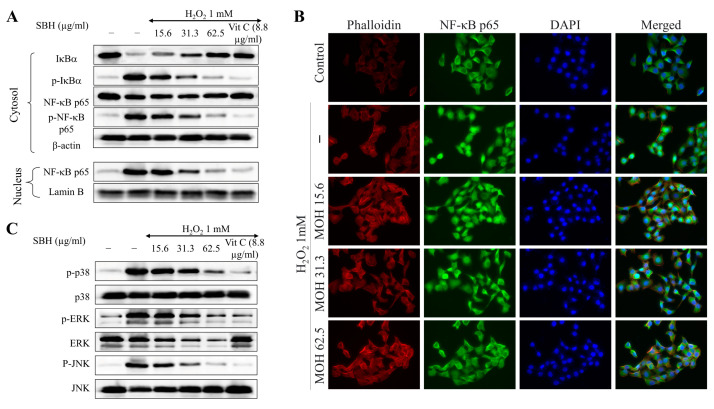
Effects of SBH in suppressing NF-κB and MAPK signaling in H_2_O_2_-induced Vero cells. Effects of SBH on NF-κB signaling pathway analyzed by (**A**) Western blot analysis and (**B**) immunofluorescence analysis of NF-κB p65 nuclear translocation; (**C**) Western blot analysis of key MAPK signaling molecules. Experiments were carried out in triplicate (*n* = 3) to ensure repeatability.

ROS both activates or represses NF-κB and MAPK signaling based on phase and context. According to the results of Western blot analysis, the H_2_O_2_ treatment increased the phosphorylation of IκBα and NF-κB p65 and the nuclear translocation of NF-κB p65 (Figure 7A). SBH treatment dose-dependently suppressed the above events, including the nuclear translocation of NF-κB p65 in Vero cells. Immunofluorescence analysis (Figure 7B) indicated a prominent green fluorescence in the nuclei of the H_2_O_2_-treated group compared to the control, indicating the nuclear translocation of NF-κB p65. The green fluorescence in the nuclei was decreased with the dose-dependent treatment of SBH, indicating the inhibition of nuclear translocation. Moreover, the SBH treatment dose-dependently lowered the phosphorylation of MAPK pathway molecules which were increased by H_2_O_2_ treatment (Figure 7C).

## 4. Discussion

Oxidative stress occurs when cellular ROS generation surpasses antioxidant capacity, contributing to the development of a number of diseases. ROS interfere with cellular signaling pathways and induce damage by reacting with essential biomolecules such as proteins, lipids (lipid peroxidation), and nucleic acids [18]. H_2_O_2_ is one of the major compounds created during oxidative stress, driven by inflammatory signals. The current study assessed the protective properties of a hot water extract of *S. borealis* and determined its bioactive principles using Vero cells that had been stimulated with H_2_O_2_.

The hot water extraction yield of *S. borealis* was recorded as 10.83 ± 0.44%. According to previous research, the 70% ethanol extract of the leaves of *S. borealis* recorded a yield of 10.4% [19]. In another study, 80% methanol room temperature extract of *S. borealis* gave a yield of 6.67% [4]. The hot water extraction method is desirable in recovering phenolic compounds considering the safety compared to the use of organic solvents. The extract indicated a higher polysaccharide and protein content with a polyphenol content of 4.82 ± 0.25%.

Phenolic constituents in *S. borealis* leaves play a major role in their bioactive properties. Previous research reported the identification of the polyphenols isoorientin, isoorientin 2-*O*-α-l-rhamnoside, tricin 7-*O*-13-d-glucopyranoside, apigenin 6-C-13-d-xylopyranosyl-8-C-13-d-glucopyranosid, (−)-syringaresinol, tricin, tricin 7-*O*-β-d-glucopyranoside, and luteolin 6-C-α-l-arabinopyranoside [4,20,21]. In the present analysis, SBH was quantified for the presence of polyphenols with reference to a known mixture of polyphenols, those which are typically found in plants. Accordingly, SBH was found to contain each of the following polyphenolic compounds within the range of 12–14% including gallic acid, 2,3,4-trihydroxybenzoic acid, 3,4-dehydroxybenzaldehyde, 4-hydroxybenzoic acid, catechin hydrate, vanillic acid, 3-hydroxybenzoic acid, chlorogenic acid, p-coumaric acid, 3,4-dimetoxybenzoic acid, sinapic acid, trans-cinnamic acid, and quercetin. Most of the aforementioned phenols are reported to process antioxidants and numerous other bioactivities [22].

Antioxidant activity of the hot water extract (SBH) was moderate for ABTS^+^ and DPPH radical scavenging activities with IC_50_ values of 96.44 ± 0.61, and 125.78 ± 4.41 µg/mL, in comparison to the positive control ascorbic acid which indicated 96.24 and 88.15% ABTS^+^ and DPPH radical scavenging activities at 10 mM concentration. In previous studies, 70% ethanol extract of *S. borealis* leaf extract indicated substantial SOD-like ability, electron-donating ability, lipid peroxidation inhibitory activity, and reducing power [23]. Per previous studies, hot water extracts of green tea (*C. sinensis*) under three different brands indicated IC_50_ values of 18.79, 35.10, and 13.51 µg/mL for DPPH radical scavenging activity and 6.81, 9.87, and 5.61 µg/mL for ABTS^+^ radical scavenging activity [24]. In comparison to green tea, antioxidant activities of SBH were low. However, these antioxidant activities can be considered moderate considering the high content of polyphenols in green tea, which is generally acknowledged for its higher antioxidant potential.

Different doses of SBH were initially evaluated for their cytotoxicity in Vero cells. Accordingly, the dose range 15.6—125 µg/mL had no toxicity and was desirable for further evaluations. The protective effects of SBH were evaluated against H_2_O_2_-induced oxidative stress by means of evaluating intracellular ROS levels and cell viability. Treatment of H_2_O_2_ increased the intracellular ROS levels in Vero cells and caused a reduction in cell viability. However, cells treated with increasing doses of SBH indicated a reduced intracellular ROS level and increased viability, suggesting that SBH possesses protective effects against H_2_O_2_-induced oxidative stress. According to a previous study, isoorientin and isoorientin 2-*O*-α-l-rhamnoside isolated from *S. borealis* have indicated strong cytoprotective effects against tert-Butyl hydroperoxide-induced oxidative damage in HepG2 cells with IC_50_ values of 9.5 and 34.5 µM, respectively [4]. Fluorescence microscopic analysis of intracellular ROS levels revealed a sudden increment of ROS levels in the H_2_O_2_-induced group, followed by a gradual and concentration-dependent decrease of fluorescence in the SBH-treated groups.

Apoptosis, also known as programmed cell death, is an essential process of cell death in multicellular organisms that help to maintain physiological conditions by mediating the removal of infected, damaged, or potentially neoplastic cells. Recent research showed that ROS and the ensuing oxidative stress play a critical role in apoptosis [25]. During apoptosis, each cell goes through an active process of cell death, which is initiated by a genetic program and results in DNA breakage and the production of membrane-packaged pieces known as apoptotic bodies. Early apoptotic cells maintain the integrity of their plasma membranes, preventing cellular material leakage. This scenario is unlike that of necrosis which proceeds via lysis of the plasma membrane. Hence, specific nuclear staining methods can distinguish between apoptotic and necrotic cells. The double staining method of Hoechst 33342 and PI applied herein is such a method that stains condensed and fragmented nuclei in apoptotic cells with a bright blue color and necrotic cells with a red color. Hoechst 33342 is a membrane-permeable nuclear stain whereas PI is impermeable. Per the outcomes, the H_2_O_2_ treatment increased the formation of apoptotic bodies as seen from nuclear condensation and fragmentation per the Hoechst 33342 staining. Moreover, few of the H_2_O_2_-treated cells indicated necrotic cell death as per the PI staining. Dose-dependent SBH treatment reduced apoptotic body formation and necrosis in the H_2_O_2_-treated Vero cells. The anti-apoptotic activity of SBH at the dose of 62.5 μg/mL was comparable with the positive control ascorbic acid at 50 µM.

Apoptosis is induced by a variety of mechanisms, including receptor-mediated signals, anti-tumor medicines, growth factor withdrawal, and DNA damage. Each of these stimuli has its own mechanism that leads to apoptotic process activation, yet they all appear to culminate in a similar sequence of events [25]. While the initial signal for apoptotic programming may vary, the morphological and biochemical characteristics of programmed cell death are uniformly similar and are highly conserved during the evolution of species. These findings suggest the interesting prospect of multiple signaling pathways that converge upstream of a common mechanism of events, leading to apoptosis. The active involvement of mitochondria is one such convergent mechanism.

The JC-1 assay was employed to evaluate mitochondrial health based on its potential-dependent accumulation in mitochondria. JC-1 produces green fluorescence at low membrane potential, whereas at high potential, the dye aggregates and produces red fluorescence. Results indicated that H_2_O_2_-treated cells contained unhealthy mitochondria (lower red and higher green) compared to the control, which is a pre-indication of the prompting apoptosis process. SBH treatment revised the effects of H_2_O_2_ making the mitochondria perform under a physiological state. The lipophilic JC-1 is a specific dye for measuring the membrane potential of mitochondria (ΔΨ(m)) [16]. Measurements with this dye offer information on changes in ΔΨ m (usually, a decrease in ΔΨ m induces a significant shift from orange to green fluorescence emission), as well as overall mitochondrial content (based on the intensity of the green fluorescence emission). A number of studies have now shown that JC-1 outperforms other dyes used for the same purpose, such as 3,3-dihexyloxadicarbocyanine iodide (DiOC6(3)) or rhodamine 123 (R123), and that JC-1 is likewise unaffected by changes in plasma membrane potential [16].

Apoptosis, which is mediated by mitochondria, is the primary mechanism of oxidative stress-induced cell death. The increase of cytochrome c, Bax, caspase-3, cleaved caspase-9, cleaved PARP, and p53 levels in H_2_O_2_-treated cells, with a decrease of Bcl2 and Bcl-xL levels, suggest the initiation of apoptosis. Moreover, PARP cleavage took place with the H_2_O_2_ treatment. The levels of apoptotic molecular mediators were dose-dependently reduced following doses of SBH, demonstrating its protective properties against H_2_O_2_-induced oxidative stress. [26]. The mitochondria-mediated apoptosis pathway is triggered by permeabilization of the outer mitochondrial membrane, which results in the release of apoptogenic molecules such as endonuclease G, cytochrome c, and apoptosis-inducing factor (AIF) found in the mitochondrial intermembranous region [27]. The activation of p53, which triggers the production of pro-apoptotic proteins, aggravates caspase-mediated apoptotic pathways. Effector caspases cleave the nuclear enzyme PARP, which is one of the most important mediators of DNA integrity, repair, and transcription [28].

Inducing Nrf2-mediated expression of HO-1 and NQO1 in cells is thought to be a viable therapeutic target for ameliorating detrimental cellular responses [29]. Nrf2 stays linked to the inhibitor Keap-1 in the cytoplasm. Nrf2 translocates into the nucleus upon stimulation, where it activates the transcription of numerous antioxidant genes, including HO-1, NQO1, glutamate-cysteine ligase, and glutathione S-transferase A2. In a variety of cell lines, HO-1 has been shown to have antioxidative, anti-inflammatory, cytoprotective, and anti-apoptotic properties. The current findings suggest that SBH can stimulate Nrf2 activation and nuclear translocation. The increase of HO-1 further clarifies the antioxidant activities of SBH. These observations collectively demonstrate that SBH increased the Nrf2 activation and therein controlled the expression of the antioxidant enzymes HO-1 and NQO1 in H_2_O_2_-stimulated Vero cells.

NF-κB p65 is a cytoplasmic transcription factor that controls gene expressions that are important for cellular processes, such as inflammatory responses, cell proliferation and apoptosis, stress responses to noxious stimuli, embryogenesis, and organ development [18]. NF-κB is generally found in the cytoplasm as a heterodimer, with the most prevalent form being p50/p65 (RelA). In these proteins, the Rel homology domain (RHD) is in charge of dimerization, recognition, and binding to DNA, as well as interaction with inhibitory κB (I-κB) proteins. Inflammatory signals cause upstream kinases (IKK) to phosphorylate I-κB proteins, resulting in the ubiquitination and destruction of I-κB. ROS is one of the factors that activates or represses NF-κB signaling based on phase and context. After that, active NF-kB translocates into the nucleus and activates the target genes. Hence, understanding the interaction between oxidative stress and NF-κB signaling is imperative for developing treatment methods for diseases in which oxidative stress plays an etiologic role. The results indicated that H_2_O_2_ treatment increased the nuclear translocation of NF-κB p65 in Vero cells, whereas SBH dose-dependently suppressed the nuclear translocation.

Further studies are being conducted to use the pink salt-brined hot water extract of *S. borealis* as a means of marinating Chub mackerel (*Scomber japonicus*, SJ) to remove the fishy smell and taste that has reduced its customer appeal. Given its antioxidant activity and the presence of desirable phytochemicals, not to mention the high growth rate and biomass, *S. borealis* could be an invaluable natural resource for manufacturing functional foods and nutraceuticals. Hence, further research could focus on a range of bioactivity evaluations for numerous diseases involving the use of in vitro and in vivo evaluations.

## 5. Conclusions

Oxidative stress has been identified as a major exacerbating factor in both acute and chronic kidney injuries. The present study investigated the effects of *S. borealis* hot water extract on hydrogen peroxide-induced oxidative stress in Vero cells. SBH reduced intracellular ROS generation. Results suggest that *S. borealis* hot water extract (SBH) is rich in antioxidants and could impose protective effects against H_2_O_2_-induced oxidative stress, reducing apoptosis. The antioxidant properties of SBH are mainly attributed to its polyphenol content. Other than the direct involvement in neutralizing ROS, metabolites in SBH were also found to induce NRF2-mediated production of the antioxidant enzymes, HO-1, and NQO1. Moreover, SBH treatment lowered NF-κB p65 nuclear translocation. These findings suggest that *S. borealis* hot water extract can be employed in producing nutraceutical and functional foods that would help to ameliorate oxidative stress-induced diseases.

## Figures and Tables

**Table 1:** Yield and proximate composition of SBH.

Sample	Yield	Proximate Composition (%)		
		Protein	Polysaccharide	Polyphenol
SBH	10.83 ± 0.44	33.09 ± 0.53	56.41 ± 0.02	4.82 ± 0.25

**Table 2:** Polyphenol composition in SBH.

Compound	Amount (µmol/100 g)
Gallic acid	79.17 ± 0.94
D-Mandelic acid	N.D.
2,3,4-Trihydroxybenzoic acid	79.29 ± 0.29
3,4-Dihydroxybenzaldehyde	97.59 ± 0.28
4-Hydroxybenzoic acid	97.74 ± 0.43
Gentisic acid sodium salt hydrate	N.D.
Catechin hydrate	46.64 ± 0.03
Vanillic acid	80.40 ± 0.35
3-Hydroxybenzoic acid	97.81 ± 2.24
Chlorogenic acid	36.86 ± 0.87
Syringic acid	N.D.
p-Coumaric acid	81.07 ± 0.36
3,4-Dimetoxy benzoic acid	70.15 ± 8.39
Sinapic acid	61.32 ± 0.75
Rutin hydrate	0.60 ± 0.37
trans-Cinnamic acid	92.06 ± 0.33
Quercetin	45.89 ± 0.06

N.D.; Not detected.

## Data Availability

Data contained in the article.
